# Mechanisms Governing Anaphylaxis: Inflammatory Cells, Mediators, Endothelial Gap Junctions and Beyond

**DOI:** 10.3390/ijms22157785

**Published:** 2021-07-21

**Authors:** Samantha Minh Thy Nguyen, Chase Preston Rupprecht, Aaisha Haque, Debendra Pattanaik, Joseph Yusin, Guha Krishnaswamy

**Affiliations:** 1 Department of Medicine, Wake Forest School of Medicine, Winston-Salem, NC 27106, USA; smnguyen@wakehealth.edu; 2 The Rowan School of Osteopathic Medicine, Stratford, NJ 08084, USA; ruppre86@rowan.edu; 3 The Bill Hefner VA Medical Center, Salisbury, NC 27106, USA; aaishahaque@gmail.com; 4 Division of Allergy and Immunology, UT Memphis College of Medicine, Memphis, TN 38103, USA; dpattana@uthsc.edu; 5 The Division of Allergy and Immunology, Greater Los Angeles VA Medical Center, Los Angeles, CA 90011, USA; Joseph.Yusin2@va.gov

**Keywords:** anaphylaxis, anaphylactic shock, hypotension, allergic reaction, angioedema, epinephrine, food allergy, mast cell, tryptase, histamine, coagulation, complement, cytokines, allergy

## Abstract

Anaphylaxis is a severe, acute, life-threatening multisystem allergic reaction resulting from the release of a plethora of mediators from mast cells culminating in serious respiratory, cardiovascular and mucocutaneous manifestations that can be fatal. Medications, foods, latex, exercise, hormones (progesterone), and clonal mast cell disorders may be responsible. More recently, novel syndromes such as delayed reactions to red meat and hereditary alpha tryptasemia have been described. Anaphylaxis manifests as sudden onset urticaria, pruritus, flushing, erythema, angioedema (lips, tongue, airways, periphery), myocardial dysfunction (hypovolemia, distributive or mixed shock and arrhythmias), rhinitis, wheezing and stridor. Vomiting, diarrhea, scrotal edema, uterine cramps, vaginal bleeding, urinary incontinence, dizziness, seizures, confusion, and syncope may occur. The traditional (or classical) pathway is mediated via T cells, Th2 cytokines (such as IL-4 and 5), B cell production of IgE and subsequent crosslinking of the high affinity IgE receptor (FcεRI) on mast cells and basophils by IgE-antigen complexes, culminating in mast cell and basophil degranulation. Degranulation results in the release of preformed mediators (histamine, heparin, tryptase, chymase, carboxypeptidase, cathepsin G and tumor necrosis factor alpha (TNF-α), and of de novo synthesized ones such as lipid mediators (cysteinyl leukotrienes), platelet activating factor (PAF), cytokines and growth factors such as vascular endothelial growth factor (VEGF). Of these, histamine, tryptase, cathepsin G, TNF-α, LTC_4_, PAF and VEGF can increase vascular permeability. Recent data suggest that mast cell-derived histamine and PAF can activate nitric oxide production from endothelium and set into motion a signaling cascade that leads to dilatation of blood vessels and dysfunction of the endothelial barrier. The latter, characterized by the opening of adherens junctions, leads to increased capillary permeability and fluid extravasation. These changes contribute to airway edema, hypovolemia, and distributive shock, with potentially fatal consequences. In this review, besides mechanisms (endotypes) underlying IgE-mediated anaphylaxis, we also provide a brief overview of IgG-, complement-, contact system-, cytokine- and mast cell-mediated reactions that can result in phenotypes resembling IgE-mediated anaphylaxis. Such classifications can lead the way to precision medicine approaches to the management of this complex disease.

## 1. Introduction

**Figure 1:**
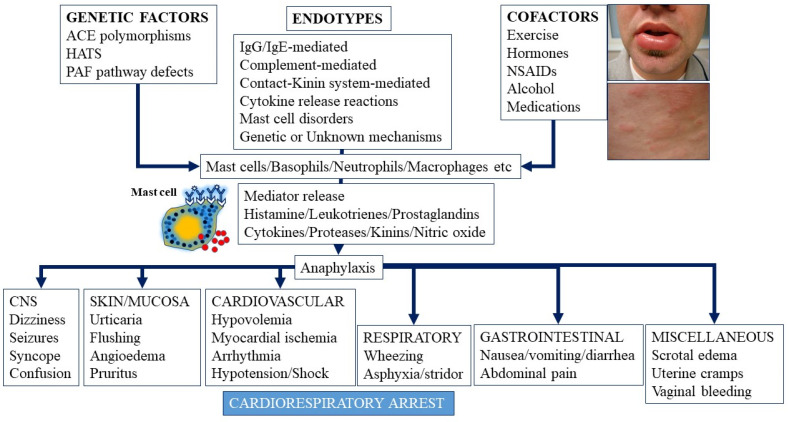
Anaphylaxis-triggers (including endotypes), genetic predisposition, selected cofactors and role of mast cells and mediators in manifestations and culminating potentially in cardiorespiratory arrest. CNS = central nervous system, NSAIDs = nonsteroidal anti-inflammatory drugs, ACE = angiotensin converting enzyme, HATS = hereditary alpha tryptasemia syndrome, PAF = platelet activating factor, IgE = immunoglobulin E, IgG = immunoglobulin G.

Anaphylaxis is a severe acute life-threatening multi-system reaction resulting from the release of a plethora of mediators from mast cells culminating in serious respiratory, cardiovascular and mucocutaneous manifestations that can be fatal [1,2]. This manifests as urticaria, pruritus, flushing, erythema, angioedema (lips, tongue, airways, periphery), myocardial dysfunction (hypovolemia, distributive or mixed shock and arrhythmias), rhinitis, wheezing, and stridor. Nausea, vomiting, diarrhea, scrotal edema, uterine cramps, vaginal bleeding, urinary incontinence, dizziness, seizures, confusion, and syncope may also be seen (Figure 1) [2,3]. Despite many advances in understanding etiology, mechanisms and management, anaphylaxis remains underdiagnosed and undertreated [4]. Anaphylaxis is highly likely when any one of the following two criteria are satisfied [1,2,5]:

1. Acute onset of an illness (minutes to hours) with involvement of the skin (urticaria, pruritus, flushing) and/or mucosa (angioedema/swelling of lips, tongue, larynx)
**Plus at Least One of the Following:**
  - **Respiratory involvement** (dyspnea, wheezing, stridor, reduced peak flows or hypoxemia);
  - **Reduced blood pressure** and associated symptoms of end-organ dysfunction (syncope, fecal or urinary incontinence, hypotonia/collapse);
  - **Severe gastrointestinal symptoms** (severe crampy abdominal pain, severe diarrhea, repetitive vomiting).
2. Acute onset of hypotension * or bronchospasm or laryngeal involvement (stridor, voice change, odynophagia) after exposure to a known or highly probable allergen for the patient even in the absence of skin involvement.

* Hypotension in adults is regarded as systolic blood pressure of <90 mm Hg or greater than a 30% decrease in systolic blood pressure from the patient’s baseline. Hypotension in infants and children: systolic blood pressure <70 mm Hg (1–12 months); <(70 mm Hg + [2 × age]) (1–10 years); <90 mm Hg (11–17 years); or >30% decrease in systolic blood pressure from baseline for the patient. 

Globally, the true rate of anaphylaxis is difficult to measure due to both under-diagnosis and misdiagnosis. It is generally agreed that the incidence of anaphylaxis is increasing worldwide with estimates for case fatality rates at 0.5–1% of patients hospitalized for the condition [6]. Anaphylaxis accounts for approximately 0.26% of all hospital admissions worldwide, with medications and food contributing to most cases [6]. Novel allergens have also contributed to the rise in anaphylaxis cases, including sensitization to mammalian meats (cross reacting with cetuximab sensitization and associated with tick bites and allergy to galactose 1,3 alpha galactose), monoclonal antibodies (biological therapies), progesterone and lipid transfer protein (a ubiquitous food allergen) [6,7,8,9]. Motosue et al. reported that over 10 years spanning 2005 to 2014, hospital admissions related to anaphylaxis increased by 37.6% [10]. According to Tejedor-Alonso and colleagues, the global prevalence of anaphylaxis is between 0.02 and 5.1% (with incidence rates averaging around 50–103 cases per 100,000 person-years) with an estimated 0.12 to 1.06 deaths per million person-years [11]. Severe anaphylaxis occurs in 1–3 per 100,000 persons with food, drugs and Hymenoptera allergy contributing to most fatal cases worldwide [12]. Recently, exercise-induced and severe food-dependent exercise-induced (FDEIA) anaphylactic reactions have been described [13]. Delayed adrenaline administration, advancing age, the use of certain antihypertensive medications and underlying cardiopulmonary disorders are factors that modify the severity of anaphylaxis and can lead to fatality [1,2,6].

Over the last decade, advances in the understanding of inflammatory mechanisms and the introduction of precision medicine led to the developments of endotype-phenotype classification of diseases such as asthma and anaphylaxis [2,14,15,16]. Using this framework, it is possible to develop more specific therapies (precision medicine) for atopic disorders [2,15]. In this review, we present recent advances in understanding mechanisms underlying severe anaphylactic reactions, including mast cell-endothelial interactions that regulate capillary permeability, and discuss the evolving areas of endotypes defining such reactions.

## 2. Materials and Methods

A search for Mesh word “anaphylaxis” yielded 30,616 results on PubMed, with 3987 papers listed under “anaphylaxis and shock”. Mesh searches were also conducted for IgG-mediated, complement-mediated, cytokine-mediated, contact-kinin-mediated and mast cell-related anaphylaxis. We chose papers published in the last 2 decades with a focus on published guidelines, systematic reviews and animal and human studies of pathophysiology-especially molecular mechanisms involving anaphylaxis. When relevant, both animal and human studies of anaphylaxis were included, realizing the inherent difficulties involved in studying molecular mechanisms in humans, especially in patients suffering from life threatening multisystem disease.

## 3. Molecular Mechanisms in Anaphylaxis: Endotypes and Phenotypes

The etiology of anaphylaxis is multifactorial in nature and can be further classified by presentation and mechanism (Table 1). Castells and coworkers describe a classification system that divides etiologies into phenotypes (clinical presentation or manifestation) and endotypes (molecular mechanisms or pathways) and this has been the subject of many recent reviews [2,14,15,16]. A phenotype has been defined as a cluster of visible properties and may include demographics, physiology (example lung function), imaging, outcomes (fatality, severity, patterns) and treatment responses (example refractory anaphylaxis) [17]. Phenotypes of anaphylaxis can be subdivided into complement-mediated, type 1-like, cytokine-storm and mixed type reactions. An endotype has been defined as “a compilation of disease mechanisms explaining disease expression within certain groups of patients”, and in the case of anaphylaxis, endotypes have included allergic/immune- (IgE or IgG-mediated), contact system-, cytokine-(cytokine release reactions or CRRs—which could be mixed due to co-occurrence of IgE-mediated reactions and cytokine storm reactions or CSRs), mast cell activation- (direct mast cell activation or clonal mast cell disorders), and complement-mediated reactions (Figure 1) [2,14,15,16,18].

It is estimated that 30–60% of patients presenting with anaphylaxis may have no obvious etiological trigger to explain the disease and hence are described as having idiopathic anaphylaxis (IA)-ultimately a diagnosis of exclusion [19,20,21]. More recently, terms such as anaphylactoid and pseudo-allergic reactions have slowly been replaced by mechanism-based descriptors [22]. The following sections discuss the mechanisms involved in such reactions in greater detail.

### 3.1. Immunoglobulin E-Dependent Anaphylaxis

Immunoglobulin E-mediated reactions to common allergens are a well-established cause of anaphylaxis (Figure 1) [1,2]. In such reactions, allergens (Table 1: Peanut/tree nuts, shellfish, egg protein, soybean, milk, latex, mammalian meat, antibiotics and other drugs, insect venom, seminal fluid, and occupational allergens) bind to specific IgE that then activates signaling pathways in mast cells and basophils expressing the high affinity receptor (FcεRI) for IgE [2,23,24,25,26]. This culminates in preformed and newly synthesized mediators from mast cells and basophils that sets off a sequence of inflammatory events manifesting clinically as anaphylaxis and leading to shock (Figure 1). Several co-factors may facilitate the evolution of severity of anaphylactic reactions, including genetic factors (deficiency of PAF-acetyl hydrolase or hereditary alpha-tryptasemia), hormones (exogenous estrogens and progesterone or perimenstrual factors), medications (lipid-lowering drugs, nonsteroidal anti-inflammatory drugs, angiotensin converting enzyme inhibitors and beta-blockers), age-related morbidities, mast cell disorders, exercise, infection and underlying cardiac, psychiatric and chronic lung disease (such as asthma) [1,2] (Figure 1). The role of mast cell signaling, the mediators expressed and their effects on vascular permeability and shock are reviewed below.

#### 3.1.1. Human Mast Cells and Basophils: Overview, Phylogeny, and Differentiation

Mast cells (MC) and basophils are prominent effector cells that are activated during IgE dependent allergic reactions and anaphylaxis [23,24,25,27,28]. Mast cells and basophils were discovered by Paul Ehrlich over 100 years ago, but recent advances have enhanced our understanding of these cell types, including their prominent roles in inflammation, autoimmunity and in allergic reactions [27,28,29,30]. The extent to which basophils contribute to anaphylaxis is debated given concomitant activation of mast cells during such process [31]. Basophils represent less than 1% of circulating leukocytes in the blood [32]. Moreover, the basophil has only a fraction of the tryptase expressed by mast cells in allergic reactions. Karasuyama, on the other hand, suggests a more prominent role for basophils in host defense, autoimmunity, and inflammation [32]. 

Human mast cells, originating from CD34^+^/CD117^+^/CD13^+^ multipotent, hematopoietic progenitors, migrate to peripheral tissues and undergo differentiation and maturation under the influence of growth factors, including stem cell factor (SCF) [23,24,25,28,33,34]. Stem cell factor is a mast cell chemoattractant that also facilitates the proliferation, differentiation, maturation, adhesion, and survival of mast cells [33]. Under the influence of local tissue-derived maturation factors, mast cell differentiation results in the development of well-recognized phenotypes-mucosal mast cells that produce only tryptase (MC_T_: located predominantly in the mucosa and alveoli and expressing cylindrical scroll granules) and connective tissue mast cells (MC_TC_: located in the skin and submucosa of the intestine and expressing lattice or grating structures) that produce chymase, tryptase, and carboxypeptidase [23,24]. Stem cell factor binds to its receptor, KIT, a transmembrane tyrosine kinase-linked receptor that enhances mast cell growth, development, and survival [23,24,25,34]. Somatic mutations in the gene (c-KIT) that codes for the KIT receptor have been linked to the development of systemic mastocytosis, a clonal hematological disorder [35,36]. The commonest of these mutations is the D816V mutation that leads to enhanced survival and proliferation of mast cells, allowing them to turn neoplastic. Mast cells undergo maturation in target tissues, a process influenced by a plethora of cytokines and growth factors expressed by resident cells or in autocrine fashion by the mast cells themselves. These include IL-3, IL-4, IL-9, IL-33, CXCL2, NGF and TGF-β1 [23,24,27,35,37,38]. Mast cell-derived tumor necrosis factor alpha (TNF-α) activates NF-kappaB in mast cells, which increases expression of GM-CSF and IL-8 from mast cells leading to a potent autocrine loop, as discussed later [38]. 

#### 3.1.2. Mast Cells: Activation and IgE-mediated Signaling

Activation of MCs occurs when allergen specific IgE is bound by allergen and interacts with FcεRI on their surfaces. Mast cells, being perivascular in location, can also extend cellular processes across vessel walls to reach luminal, circulating specific IgE. The production of allergen-specific IgE involves antigen processing cells, T cell subsets and B lymphocytes, but the exact pathways are unclear [39]. The exposure of B lymphocytes to food allergens likely occurs in the stomach and duodenum and associated lymphoid tissue. Class switch recombination of B lymphocytes occurs locally in the gastrointestinal tract mucosa and allows the expression of IgE [39]. Food allergens can also be processed by dendritic cells that migrate to the regional lymph nodes where corresponding antigens are presented to naïve T lymphocytes in an MHCII restricted fashion. Naïve T cells differentiate subsequently into Th2 and T follicular helper cells (TFH) capable of expressing a distinctive set of cytokines, including IL-4, IL-5 and IL-13 that induce IgE class switch recombination in B cells. B cells differentiate under the influence of cytokines or on contact with TFH into IgE-secreting plasma cells and this IgE binds to the corresponding receptor on mast cells [39]. Besides receptors for IgE (FcεRI), human mast cells express receptors for IgG (FcγRII/III), Complement (C3a/C5a), drugs (MRGPRX2), opioids, neuropeptides, nerve growth factor, stem cell factor and cytokines, ligation of which modify mast cell function-survival, maturation, differentiation, growth, apoptosis, and degranulation [22,23,28]. Dendritic cells sampling the lumen of blood vessels and their utilization of microvesicles to trigger mast cell degranulation and anaphylaxis were demonstrated recently in a murine model of anaphylaxis. Hence, luminal sampling of antigens and/or IgE is a feature of both mast cells and dendritic cells, and can lead to more efficient antigen presentation and cellular activation under these circumstances [40].

**Figure 2:**
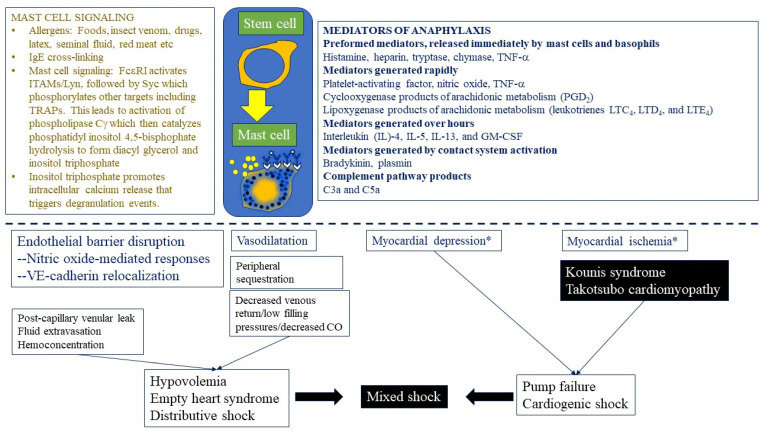
Human MCs, originating from CD34^+^/CD117^+^/CD13^+^ multipotent, hematopoietic progenitors, migrate to peripheral tissues and undergo differentiation and maturation under the influence of growth factors, including stem cell factor (SCF). The figure shows mast cell signaling and mediators expressed in anaphylaxis and their potential relationship to endothelial, blood vessel, myocardial and circulatory effects. A complex sequence of events including endothelial barrier disruption, fluid extravasation, vasodilatation, decreased venous return, myocardial depression, and myocardial ischemia lead to distributive or mixed shock pictures. ITAM = immunoreceptor tyrosine-based activation motifs; Lyn/Syc/TRAPS/PLC/PIP2/DAG/IP3 are signaling molecules (see text for elaboration). CO = cardiac output.

The high affinity receptor for immunoglobulin E, FcεRI, has a tetrameric structure composed of an α chain which binds to IgE, a β chain signaling subunit, and two γ subunits existing as homodimers containing the immunoreceptor tyrosine-based activation motif (ITAM) [41]. The ℽ chain is essential for signal transduction and mast cell activation, while the β chain has been shown to amplify the signal induced by ℽ chain [23,41]. The role of IgE antibody in triggering anaphylaxis in an allergen-specific manner has been well established [27,42]. The cross-linking of FcεRI through IgE-bound allergen (Figure 2) leads to influx of extracellular Ca^++^ which is required for the release of preformed and newly generated mediators as well as several Ca^++^ -dependent cytokines [23,24,25,28,43]. The signaling pathways leading to the release of calcium have been characterized over the last two decades. Binding of the allergen and IgE to the high affinity receptor for immunoglobulin E, FcεRI, activates ITAMs/Lyn, followed by Syc which phosphorylates other targets including TRAPs (Figure 2). This leads to activation of phospholipase Cγ(PLCγ) which then catalyzes PIP_2_ (phosphatidyl inositol 4,5-bisphophate) hydrolysis to form DAG (diacyl glycerol) and IP_3_ (inositol triphosphate). The signaling molecule, IP_3_, promotes intracellular calcium release that triggers degranulation (Figure 2). 

#### 3.1.3. Mast Cell Degranulation

During degranulation certain unique events occur, and mast cell granules fuse with lysosomes to form secretory lysosomes. Granule exocytosis can occur by one of several techniques-multivesicular or sequential exocytosis and by piecemeal degranulation involving gradual loss of granule content [23]. Membrane retrieval occurs by a process of endocytosis, also by multiple techniques-clarithrin invagination, kiss-and-run endocytosis, and bulk endocytosis [23]. Degranulation of preformed mediators (histamine, heparin, tryptase, chymase, carboxypeptidase, cathepsin G and TNF-α), is followed by the release of de novo synthesized mediators such as lipid mediators (cysteinyl leukotrienes-LTC_4_, LTD_4_, LTE_4_), PAF, cytokines (TNF-α, GM-CSF, IL-1, IL-3, IL-5, IL-4/IL-13, IL-6, IL-10), chemokines (such as CCL-2, CC-3, CCL-5, CXCL-8) and growth factors (such as transforming growth factor beta 1 (TGF-β1), stem cell factor (SCF) and vascular endothelial growth factor [VEGF]) (Figure 2) [23,24,25,28,44]. Of these, histamine, tryptase, cathepsin G, TNF-α, LTC_4_, PAF and VEGF appear to be capable of increasing vascular permeability [45].

#### 3.1.4. Pivotal Mediators Expressed by Mast Cells and Basophils

Histamine. released from mast cells and basophils following degranulation, is a major mediator of anaphylaxis. Experimental intravenous infusion of histamine in healthy volunteers can reproduce many of the symptoms and signs of anaphylaxis: flushing, headache, wheezing and transient hemodynamic changes such as systemic hypotension and tachycardia [31]. Histamine can bind to 4 types of receptors: H_1_ (binding of histamine generates nitric oxide), H_2_ (present on Th_1_ lymphocytes and mediating acid secretion, Th_1_ cytokine synthesis and regulation of vascular permeability), H_3_ (modulating blood–brain barrier function and regulation of cognition and sleep–swake cycles) and H_4_ (modulating cytoskeletal rearrangement, eosinophil recruitment and cell adhesion molecule expression) [46,47]. Histamine binding to H_1_ receptor causes increased vascular permeability, bronchospasm, and GI contraction. Histamine binding to H_2_ receptor leads to flushing, glandular secretion, and cardiac contraction. 

Cysteinyl leukotrienes (CysLTs such as LTB_4_, LTC_4_ and LTD_4_) are synthesized following degranulation of mast cells and basophils and promote acute allergic reactions by increasing permeability of endothelium, enhancing vasodilatation, and recruiting inflammatory cells [31,45,48]. Arachidonic acid is first converted into leukotriene A_4_ by 5-lipoxygenase which then undergoes conversion to LTC_4_ by LTC_4_ synthase (LTC_4_S) [45]. LTC_4_ is subsequently converted to the other CysLTs, LTD_4_ and E_4_ in the extracellular environment [45]. Cysteinyl leukotrienes appear to be much more potent than histamine with regard to their vascular effects. The evidence regarding the role of CysLTs in anaphylaxis comes from mouse models. Using passive transfer IgE mediated cutaneous anaphylaxis mice models, investigators have shown that the severity of anaphylaxis is reduced when mice lack LTC_4_S and/or Cys-LT receptors [49,50]. In healthy volunteers, intradermal injection of CysLTs elicit wheal and flare reactions and aerosol administration causes potent bronchoconstriction [31]. Leukotrienes have been implicated in the late fall in coronary blood flow and have been implicated in the prolonged contractile failure in cardiac anaphylaxis [51].

The role of PAF as a potent mediator of anaphylaxis has been established in mouse models of anaphylaxis as well as in human subjects [52]. The platelet activating factor binds to the PAF receptor on various cells, such as endothelial cells (signaling pathways are discussed later), platelet, monocytes, macrophages, and neutrophils leading to increased vascular permeability, bronchial smooth muscle contraction, circulatory collapse and decrease cardiac output [52]. The enzyme, PAF-Acetyl Hydrolase (PAF-AH) degrades PAF into inactive lysoPAF and regulates the half-life of the mediator. Levels of PAF are elevated in anaphylaxis and severity of anaphylaxis has been correlated with a higher level of PAF [53,54]. The severity of anaphylaxis has also been inversely correlated with a lower level of PAF-AH activity [53,54]. Brown et al., described several risk factors for patients who developed severe reactions-older age, pre-existing lung disease and drug-related anaphylaxis [54]. Severe reactions were associated with low platelet activating factor acetyl hydrolase (PAH) and elevated levels of mediators such as histamine, tryptase and cytokines. Levels of mast cell tryptase, histamine, IL-6, IL-10, and tumor necrosis factor receptor 1 were also associated with delayed deteriorations (majority within 4 h of initial epinephrine administration) [54]. The platelet activating factor is the most potent mast cell mediator identified to date and is associated with decreased cardiac output, vascular hyperpermeability, smooth muscle contraction, hypovolemia, and cardiac collapse [52,55]. A deficiency of PAF acetyl-hydrolase could lead to more severe anaphylaxis in a PAF-dependent manner [53,56]. Mediators such as prostaglandin D_2_ (PGD_2_), tryptase and other cytokines and chemokines released from mast cell and basophils during anaphylaxis can also exacerbate anaphylaxis [31]. 

#### 3.1.5. Role of Mast Cell Mediators in Anaphylaxis

Mast cell-derived cytokines, histamine, leukotrienes, prostanoids and PAF regulate vascular instability, barrier dysfunction of endothelial cells and contribute to edema formation [57]. Histamine specifically can contribute to headache, nausea, pruritus, flushing, gastric hypersecretion, nasal congestion, and wheezing [57]. Histamine, leukotrienes and PAF may be responsible for pulmonary edema and gastrointestinal symptoms such as cramping and diarrhea. Histamine, PAF and kinins such as bradykinin, may contribute to angioedema. Histamine, PGD2, leukotrienes, PAF and prostanoids are responsible for wheezing and mucus hypersecretion while cytokines, chemokines, leukotrienes, and histamine may lead to neurological symptoms including headaches, fatigue, sense of impending doom and confusion [57]. Studies suggest that activation of the contact system and secretion of plasminogen activator and heparin may lead to coagulation abnormalities and bleeding seen in some patients.

#### 3.1.6. Mast Cell-Mediated Paracrine, Endocrine, and Autocrine Loops 

**Figure 3:**
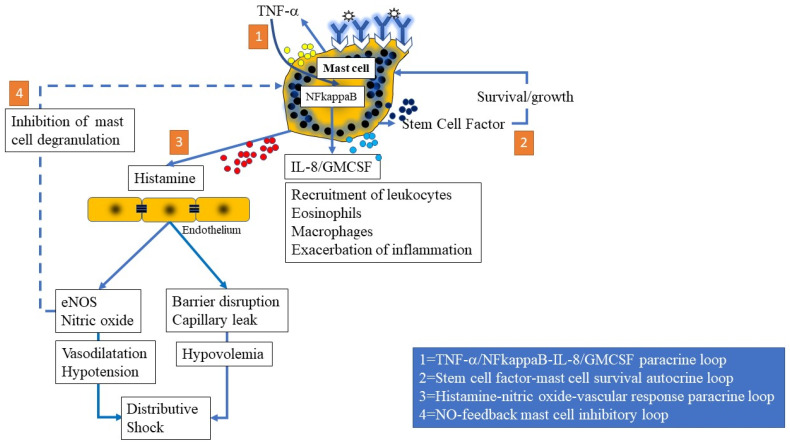
Multiple autocrine and paracrine loops that may play a role in refractory anaphylaxis. 1 = TNF-NFkappaB loop; 2 = Stem cell factor-mast cell growth loop; 3 = mast cell histamine-endothelial nitric oxide synthase (eNOS or NOS3) and barrier disruption loop; 4 = Feedback inhibition of mast cell degranulation by NO.

Specific mediators released by mast cells may have autocrine, paracrine, and endocrine effects of interest to the pathogenesis of anaphylaxis (Figure 3). The monokine and mediator of the acute phase response, TNF-α, is produced by mast cells and can, by activating its receptors, induces the autocrine expression of nuclear factor kappaB (NFκB) in mast cells. Inhibition of autocrine TNF-α effects using blocking antibodies significantly reduced NFκB activation by anti-IgE. It therefore appears likely that the release of preformed mast cell-associated TNF-alpha acts as a positive autocrine feedback signal to enhance NFκB activation [38]. This transcription factor can induce the expression of IL-8 and GM-CSF from mast cells, leading to expansion of the inflammatory cascade [38]. The cytokines IL-8 and GM-CSF, in turn, have paracrine effects on other cells of leukocyte lineage, including eosinophils, neutrophils and macrophages. Similarly, the expression of SCF by mast cells can have facilitatory autocrine effects on mast cell survival, chemotaxis, growth, and proliferation [37]. Another paracrine loop seen following mast cell activation is mast cell-derived histamine binding to endothelial H1-receptors and leading to the elaboration of NO [58,59,60]. Interestingly, a remote autocrine loop is created by histamine-induced NO, which has net inhibitor effects on mast cell activation, degranulation, mediator expression and cytokine secretion [61]. Remote effects of NO and mast cell-derived TNF-α and prostaglandins on hypothalamopituitary axis function may represent an endocrine-type response.

#### 3.1.7. The Classical IgE-histamine Pathway versus the IgG-FcγR-PAF Pathway

From studies in murine models, it has been suggested that mast cell or basophil-derived histamine and PAF enhance nitric oxide production from the endothelium, which mediates the increased vascular permeability and resulting capillary leak, hypovolemia, and hypotension [62,63,64]. There are 2 murine models of anaphylaxis-passive systemic anaphylaxis (PSA) and active systemic anaphylaxis (ASA) [65]. The passive systemic anaphylaxis model involves naïve mice passively infused with allergen-specific IgE followed by parenteral administration of the relevant allergen [65]. When mast cell, histamine (histamine synthase) or IgE deficient mice are used, anaphylaxis is totally abrogated, and vascular hyper-permeability and hypotension are not evident [66,67]. This is referred to as the classical pathway and involves the IgE-FcεRI-mast cell axis. In the ASA model, on the other hand, the allergen that is used to first sensitize the mouse is also used for a subsequent challenge. This leads to a more robust IgG response rather than an IgE-mediated one, with more severe and fatal anaphylaxis outcomes in murine models. In this model, when mast cell-, histamine- (histamine synthase deficiency) or IgE-deficient mice are used, anaphylaxis is not completely abrogated. Vascular hyper-permeability and hypotension are still evident, and fatality still occurs. However, elimination of the IgG receptor, FcγR, leads to resolution of anaphylaxis and reversal of fatality. Inhibition of the PAF receptor leads to similar effects, suggesting that an alternative pathway of anaphylaxis exists, dependent on an IgG/Fcγ/PAF axis [65]. Compared to the classical pathway, which is seen in most allergic reactions (to foods, drugs, venom, latex etc), the alternative pathway is demonstrable in only a few circumstances in humans (Table 1). 

#### 3.1.8. Endothelial Cell–Cell Adhesions: Structure and Function

**Figure 4:**
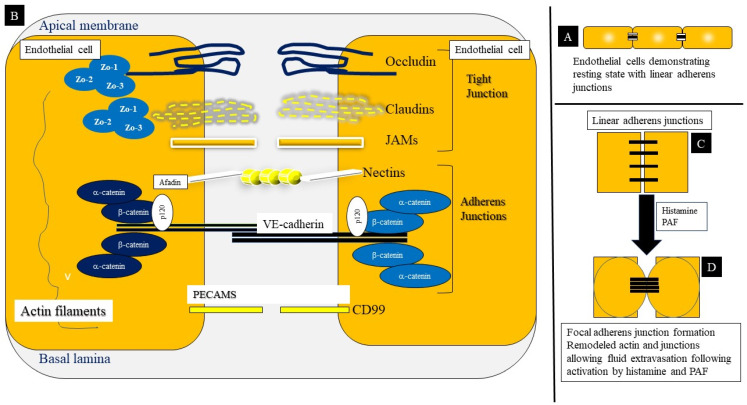
Structure of the adherens and tight junctions in endothelial cell–cell contact and barrier development. (**A**) This represents resting state with AJ configuration regulated by mechanical forces and shear stress. (**B**) The endothelial barrier is regulated by a set of cell–cell adhesions which include tight junctions (TJs), adherens junctions (AJs), gap junctions and other molecules (including nectins, PECAM and CD99) which in turn associate with the actin skeleton. Three types of catenins (α, β and γ [plakoglobin]) bind to and stabilize the VE-cadherin molecule, with α-catenin serving as a communication of cadherins with actin filaments. (**C**) The VE-cadherin-catenin complex also associates with vinculin (V) which serve as actin-binding protein. 120-Catenin binds directly to the cytoplasmic domain of VE-cadherin, close to the membrane, while the β- and γ-catenins bind to the cytoplasmic tail and helping to anchor α-catenin which in turn is tethered to the actin cytoskeleton by other proteins including vinculin, α-actinin and afadin. (**D**) Permeability-increasing factors such as histamine and PAF cause remodeling of the actin cytoskeleton and destabilization of the endothelial cell to cell junctions. Induction of radial contractile actin bundles, and associated actin-myosin contraction causes the re-localization of linear VE-cadherin complexes to focal adherence junctions leading to formation of intercellular gaps in endothelial cells and resultant hyperpermeability.

Endothelial cells control the transport of fluids, inflammatory cells, and solutes as well as mediators across the blood vessels into tissue and interstitial fluid (Figure 4A) [68]. The endothelial barrier is regulated by a set of cell–cell adhesions which include tight junctions (TJs), adherens junctions (AJs), gap junctions and other related molecules (including nectins, PECAM and CD99) which in turn associate with the actin skeleton [68]. The junctional complexes regulating the endothelial cell barrier are shown in Figure 4B and are essential to understanding the effects of histamine and PAF in anaphylaxis [69]. 

Adherens junctions are protein complexes forming cell–cell adhesive interactions and being linked to the actin cytoskeleton using their cytoplasmic components, they connect the cytoskeleton of adjacent cells [69]. The central component of the AJ is vascular endothelial cadherin (VE-cadherin) which forms contacts by means of homophilic interactions of its extracellular domains [69]. The vascular endothelial cadherin influences endothelial cell permeability, leukocyte migration and blood vessel morphogenesis [70]. Three types of catenins (α, β and γ[plakoglobin]) bind to and stabilize the VE-cadherin molecule, with α-catenin serving as a communication of cadherins with actin filaments (Figure 4B). The vascular endothelial cadherin-catenin complex also associates with vinculin which serve as an actin binding protein and demonstrates continuous staining on light microscopy. These represent resting or stable AJs (Figure 4C). 120-catenin binds directly to the cytoplasmic domain of VE-cadherin, close to the membrane, while the β- and γ-catenins bind to the cytoplasmic tail and help to anchor α-catenin. This in turn is tethered to the actin cytoskeleton by other proteins including vinculin, α-actinin and afadin. Permeability-increasing factors such as histamine and PAF cause remodeling of the actin cytoskeleton and destabilization of the endothelial cell to cell junctions. Induction of radial contractile actin bundles, and associated actin-myosin contraction causes the re-localization of linear VE-cadherin complexes to focal adherence junctions (Figure 4D) leading to formation of intercellular gaps in endothelial cells, culminating in hyperpermeability [69]. Permeability enhancers such as TNF-α, on the other hand, promote microtubule depolarization that increase permeability by unclear mechanisms [69]. 

Tight junctions (Figure 4B) are more commonly found in brain microvasculature and composed of several proteins such as claudins, occludins, junctional adhesion molecules (JAMs) and endothelium-specific adhesion molecules (ESAM), which interact with several scaffolding zona occludens proteins, cingulin and paracingulin. Blood flow mediates stabilization of TJs via the recruitment of Notch1 and TRIO which then activate protein kinase A (PKA) and Rac1 in sequence. This is followed by the stabilization of the endothelial barrier, decreasing permeability. Mutations in Notch1 lead to perturbations in endothelial gap junctions [70]. Gap junctions are made up of connexin proteins and allow passage of water, small ions, and small molecules. The roles of TJs and gap junctions in anaphylaxis are unknown. 

#### 3.1.9. Endothelial Barrier Dysfunction in Anaphylaxis

The endothelial barrier can be disrupted by a plethora of factors released during anaphylaxis or during infectious or inflammatory states. These permeability-enhancing factors include TNF-α, IL-6, histamine, PAF, kinins and reactive oxygen species. Activated mast cells in anaphylaxis can express histamine, TNF-α, PAF and VEGF, all of which have been shown to increase vascular permeability by influencing function of AJs [65].

**Figure 5:**
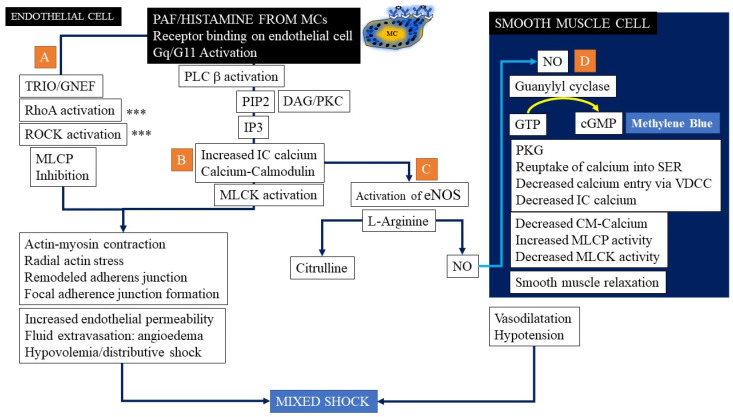
Binding of PAF and histamine to their respective receptors which are G protein-linked leads to activation of G_q_/G_11_. This leads to the activation of the guanine nucleotide exchange factor, Trio, which in sequence activates the small GTPases such as RhoA, which then activate the serine/threonine kinase, ROCK which in turn phosphorylates myosin light chain phosphatase (MLCP), inhibiting its activity (**A**). Receptor binding activates phospholipase Cβ (**B**) which then catalyzes PIP_2_ (phosphatidyl inositol 4,5-bisphophate) hydrolysis to form DAG (diacyl glycerol) and IP_3_ (inositol triphosphate). Calcium-dependent activation of MLC kinase (MLCK) now occurs, resulting in increased acto-myosin contractility and contributing to changing actin bundle orientation (induction of radial actin stress fibers) with the latter switching from being parallel to the junctions to perpendicular, thereby inducing junctional stress and disrupting integrity and vascular leakiness. Meanwhile, nitric oxide (NO), produced not by inducible nitric oxide synthase (iNOS), but by constitutive endothelial form (eNOS) that is rapidly activated via the PI3K/Akt pathway (**C**). NO induces the formation of cGMP in smooth muscle cells from sGC (**D**), which then activates PKG leading to a reuptake of calcium from the cytosol by the sarcoplasmic reticulum (SER) as well diminished calcium influx via voltage-dependent calcium channels (VDCC). This, combined with the opening of potassium channels and the exit of calcium out of the cell leads to drop in intracellular calcium concentrations, inactivation of calmodulin and resultant failure to activate MLCK. MLC phosphatase activity also increases correspondingly, leading to disruption of the actin-myosin cross-bridge and causing vasodilatation of blood vessels. Methylene blue has become a novel treatment for refractory cases of distributive shock due to its inhibitory effects on the eNOS-cGMP pathway.

Recently, mechanisms underlying histamine- and PAF-induced changes in vascular permeability have been studied in greater detail using laboratory models (summarized in Figure 5) [65]. Histamine and PAF can regulate vascular permeability as well as induce vasodilatation. Ashina et al. reported how NO functions synergistically with histamine to enhance vascular permeability [71]. Binding of histamine or PAF to their respective receptors, which are G protein-linked, leads to activation of G_q_/G_11_, which then activates phospholipase C (PLCβ) [64,72]. Experiments in animal models have demonstrated a pivotal role for G proteins in histamine-induced generation of NO [72]. Genetic mouse models with Gq/G11 knocked out have demonstrated that signaling via G proteins is crucial to enhancing vascular permeability as well as in inducing eNOS in endothelial cells [72]. Korhonen and colleagues demonstrated that knocking out endothelial G proteins linked to histamine and PAF receptors led to blunting of anaphylaxis in their mouse model [72]. Mikelis et al., showed the roles played by small Rho GTPases and ROCK in anaphylactic vascular permeability in genetically modified mice [73]. This was confirmed by Kugelmann and coworkers who demonstrated disruption of AJs with histamine was mediated by a calcium-Rhoa-ROCK pathway [74]. Bradykinin, histamine, and PAF-induced activation of these G proteins via their respective receptors probably leads to the activation of the guanine nucleotide exchange factor, Trio, which in sequence activates the small GTPases such as RhoA, which then activate the serine/threonine kinase, ROCK (Figure 5 Panel A). This in turn phosphorylates myosin light chain phosphatase (MLCP), inhibiting its activity [69]. This leads to radial actin fiber stress and remodeling of adherens junctions contributing to the formation of focal adherens junctions and the development of holes in the endothelial barrier. This disruption of the tight barrier leads to extravasation of fluids to the extravascular space rapidly in anaphylaxis. The fluid deficits in patients suffering from anaphylactic shock have been shown to be quite large, requiring rapid resuscitation using wide bore catheters or central lines. 

Another pathway can be activated by PAF and histamine binding to their respective receptors. This activates phospholipase Cβ (Figure 5B) which then catalyzes PIP_2_ (phosphatidyl inositol 4,5-bisphophate) hydrolysis to form DAG (diacyl glycerol) and IP_3_ (inositol triphosphate). Calcium-dependent activation of MLC kinase (MLCK) now occurs, resulting in increased acto-myosin contractility and contributing to changing actin bundle orientation (induction of radial actin stress fibers) with the latter switching from being parallel to the junctions to perpendicular, thereby inducing junctional stress and disrupting integrity and vascular leakiness [68]. Other mechanisms may play a role, and endothelial cell apoptosis (seen with TNF-α) as well as phosphorylation of members of the cadherin-catenin complex can occur, resulting in disassembly of the complex, contributing to leakiness [75]. The role of endothelial cell apoptosis has not been evaluated in anaphylaxis but has been shown to be of possible importance in inflammatory and immune disorders of diverse etiologies. Cytokines such as TNFα, endotoxin, shear stress and hypoxemia may contribute to endothelial apoptosis in anaphylaxis and sepsis, but experimental or clinical data are lacking making it difficult to propose any significant pathological role for apoptosis in vascular leakiness. The same calcium-calmodulin complex that activates endothelial barrier disruption can go on to activate the nitric oxide pathway and this is discussed in the next section (Figure 5C).

#### 3.1.10. Nitric Oxide Pathway Activation in Anaphylaxis 

**Figure 6:**
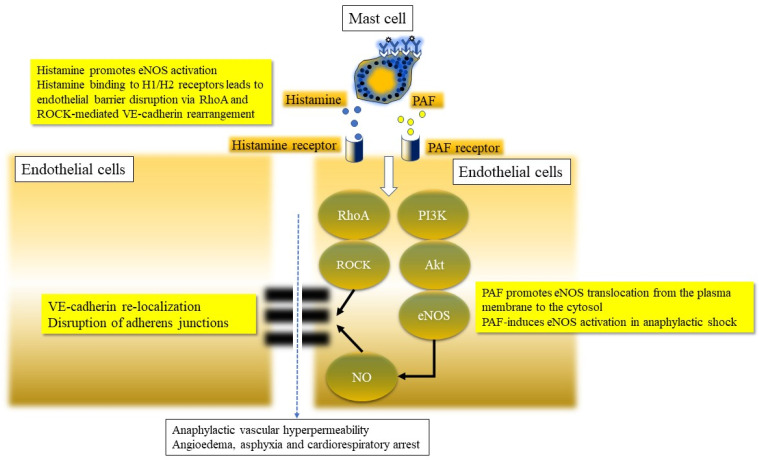
Activation pathways of histamine and PAF with involvement of their respective endothelial receptors are shown-leading to capillary permeability changes via vascular endothelial-cadherin (VE cadherin) and adherens junction (AJ) disruption. This culminates in fluid leak from postcapillary venules (PCV) contributing to hypovolemic/distributive shock. The elaboration of other mediators can also contribute to bronchospasm, angioedema and respiratory distress leading to asphyxia/hypoxemia and respiratory arrest. Involvement of RhoA and ROCK signaling in VE-cadherin relocalization as well as the PI3kinase-Akt pathway of eNOS activation are shown in cartoon format (adapted from Nakamura and Muraata, 2018) [65].

Another mechanism involved in both vasodilatation, as well as increased endothelial leaking, is related to the production of nitric oxide (NO) within endothelial cells (Figure 5C). Histamine as well as PAF can lead to activation of eNOS contributing to the formation of NO via a PI3Kinase-Akt pathway (Figure 5C,D and Figure 6). The study in mice by Cauwels and colleagues suggested that endothelial nitric oxide synthase (eNOS) plays a pivotal role in anaphylaxis [62,63]. The authors state that hyperacute PAF shock depended entirely on NO, produced not by inducible nitric oxide synthase (iNOS), but by constitutive endothelial form (eNOS), rapidly activated via the PI3K/Akt pathway (Figure 6). In 2 different models of active systemic anaphylaxis, inhibition of NOS, PI3K, or Akt or eNOS deficiency provided complete protection. The mediator, IP, generated by histamine and/or PAF binding to their respective receptors, has been shown to release cytosolic calcium from the endoplasmic reticulum. Calcium binds calmodulin, and this complex activates eNOS [64]. The activation of eNOS leads to the production of NO from the substrate, arginine, leading to VE-cadherin relocalization to focal adhesive junctions via a Rhoa-ROCK pathway (Figure 6). 

The effect of diffused NO on neighboring smooth muscle cells also triggers vasodilatation (Figure 5D and Figure 6) [61,69,76]. Nitric oxide induces the formation of cGMP in smooth muscle cells from soluble guanylyl cyclase abbreviated as sGC (Figure 5D), which then activates protein kinase G (PKG) leading to reuptake of calcium from the cytosol by the sarcoplasmic reticulum as well diminishing calcium influx via voltage-dependent calcium channels (VDCC) [77,78,79]. This, combined with the opening of potassium channels and the exit of calcium out of the cell leads to a drop in intracellular calcium concentrations, followed by inactivation of calmodulin and resultant inability to activate myosin light-chain kinase. MLC phosphatase activity also increases correspondingly, leading to disruption of the actin-myosin crossbridge. This promotes vascular dilatation, and the combination of vasodilatation and capillary leak with fluid extravasation as well as any superimposed cardiac depression culminates in a picture of mixed or distributive shock in anaphylaxis (Figure 5).

**Figure 7:**
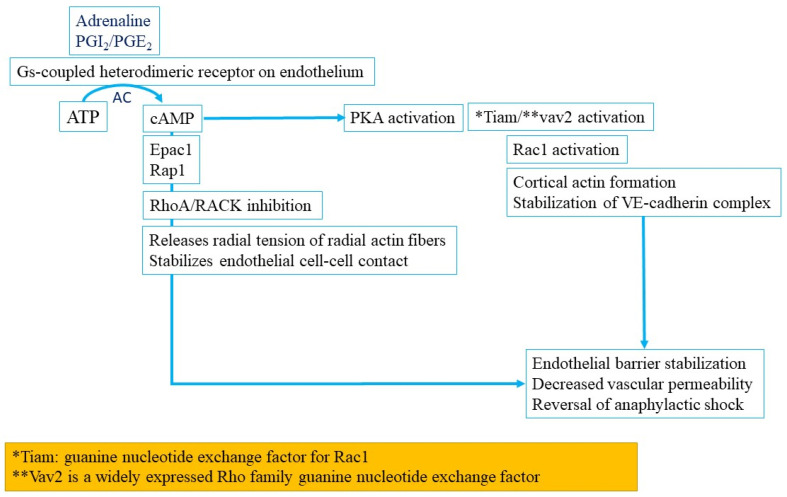
Epinephrine (or adrenaline), prostacyclin (PGI2) and PGE2 bind to G protein linked receptors, which activate adenyl cyclase (AC), which in turn leads to the formation of cyclic AMP (cAMP). cAMP via Epac1 activates Rap1 which inhibits Rhoa and ROCK and thereby relieves the radial tension of the radial actin fibers. Another pathway via Protein kinase A (PKA)/Tiam-Vav2-Rac1 pathway results in stabilizing VE-cadherin complexes.

Epinephrine (adrenaline) and PGE_2_, unlike histamine and PAF, have protective effects on the development of anaphylaxis and their mechanisms of action in IgE-mediated anaphylaxis are now better understood (Figure 7) [69]. Both epinephrine (or adrenaline) and PGE_2_ bind to G protein-linked receptors, which activate adenyl cyclase. This in turn leads to the formation of cyclic AMP (cAMP) which via Epac1 activates Rap1 leading to inhibition of RhoA and ROCK. The resulting release of the radial tension of actin fibers helps stabilize the hyperpermeability of the endothelial cell layer [69]. The molecule, Rap1, also stabilizes cell–cell contact regions by recruiting a host of other proteins in a complicated signaling cascade and strengthening VE-cadherin junctional integrity. Methylene blue has become a novel treatment for refractory cases of distributive shock due to its inhibitory effects on the eNOS-cGMP pathway and has a different mechanism of action in anaphylactic shock [80]. Methylene blue exerts its therapeutic effect by inhibiting the nitric oxide cGMP pathway through selective blocking of guanylate cyclase [80]. Halting guanylate cyclase leads to the reversal of systemic vasodilation.

### 3.2. IgG-Mediated Anaphylaxis

These reactions are mediated by IgG-immune complexes binding to the low affinity receptor, FcγRIII, on macrophages, and activating PAF synthesis. Platelet activating factor is a product of mast cells but can also be elaborated by activated platelets, neutrophils, and monocytes. It can activate platelet aggregation, induce the release of thromboxanes and serotonin and lead to endothelial permeability and capillary leak and vasodilatation. Decreased cardiac output and bronchospasm or diarrhea have also been attributed to PAF [81]. Direct evidence for the existence of IgG-mediated anaphylaxis is lacking in humans, though it is believed to be operational in selected clinical situations [26,82,83,84]. Murine studies have demonstrated that low dose antigen may stimulate a blocking IgG response that prevents IgE-mediated anaphylaxis from occurring whereas large doses of antigen could potentially mediate IgG-mediated anaphylaxis [16,26]. Some probable examples of IgG-mediated anaphylaxis include reactions to infusions of biological agents (example infliximab), dextrans, intravenous immunoglobulin (in patients with IgA-deficiency), aprotinin, Von Willebrand factor infusions and other medications [14,16,85,86,87,88,89]. In one study by Jannsson et al. the authors prospectively evaluated 86 patients with suspected anaphylaxis to neuromuscular-blocking agents (NMBAs) during general anesthesia and 86 matched controls [86]. The authors report on the presence of anti-NMBA IgG and markers of FcγR activation, PAF release, and neutrophil activation, all of which correlated with the severity of anaphylaxis. In this elegant study, the authors showed that neutrophils underwent degranulation and NETosis early after anaphylaxis onset. Moreover, anti-NMBA IgG triggered neutrophil activation ex vivo in the presence of NMBA and neutrophil activation could also be observed in patients lacking evidence of classical IgE-dependent anaphylaxis. Neutrophils in such situations may be recruited in anaphylactic reactions [90]. Accordingly, in all these reported cases, large doses of antigen were infused, allowing a shift to an IgG-mediated reaction rather than an IgE-mediated one. 

### 3.3. Cytokine Release and Cytokine Storm Reactions

Cytokine release reactions (CRRs) or cytokine storm reactions (CSRs) can be induced by certain monoclonal antibodies (chimeric, humanized and fully human) or by chemotherapeutic agents [14,16,91,92,93]. Cytokine release-related reactions are probably mediated by tumor necrosis factor alpha (TNFα), IL-1β and IL-6 produced by mast cells, monocytes, and T cells. Such reactions can present with fever, chills, and pain, responding to the administration of nonsteroidal drugs and rigorous hydration. A more severe variant of this reaction is called cytokine storm, characterized by expression of larger quantities of cytokines (IL-8, IFNγ, TNFα, IL-1β and IL-6), vascular leakage related to increased endothelial permeability, activation of thromboplastin and the coagulation system leading to a multisystem syndrome characterized by organ failure and disseminated intravascular coagulation (DIC). Fever, chills, hypotension, hypoxemia, and cardiovascular collapse rapidly ensue, requiring urgent intervention and management in an intensive care unit [15,81]. The onset of CRS is usually 2 to 3 days after infusion of medications, with flu-like symptoms (fever, anorexia, myalgia, headaches, rigor, malaise) followed by either spontaneous resolution or progression to multisystem involvement with hypotension, capillary leak, pneumonitis, hypoxemia, renal insufficiency and disseminated intravascular coagulation [91,94]. 

Severe CRS may be associated with cardiopathy, low ejection fraction and elevated troponins and can be fatal if not aggressively managed. Inflammatory markers, especially ferritin and C-reactive protein levels, are elevated and findings consistent with macrophage activation syndrome (MAS) or hemophagocytic lymphohistiocytosis (HLH) may be seen. It has been postulated that IL-1 released from monocytes and macrophages activates endothelial expression of NO and IL-6, which in turn induce an acute phase response complicated by the systemic inflammatory response syndrome (SIRS) [94]. Coagulopathy occurs due to the release of tissue factor and platelet endothelial cell adhesion molecule-1 (PECAM-1). Reactive oxygen species may further stimulate the production of pro-inflammatory cytokines by activating nuclear factor kappaB, leading to a vicious inflammatory cycle. Cellular activation, inflammatory response, widespread coagulation abnormalities, and ischemia-reperfusion injury may be seen in cytokine storms [91]. Glucocorticoids, hemadsorption (of inflammatory cytokines), hyperbaric oxygen, anti-cytokine therapies (anti-IL1, anti-IL18, anti-TNFα and anti-IL6) as well as JAK-STAT inhibitors (especially baricitinib) have been used to reverse cytokine toxicity [91]. In some studies, mast cell stabilizers such as sodium cromoglycate or ketotifen have shown some benefits but are not yet considered standard therapy [91].

### 3.4. Contact System Activation

**Figure 8:**
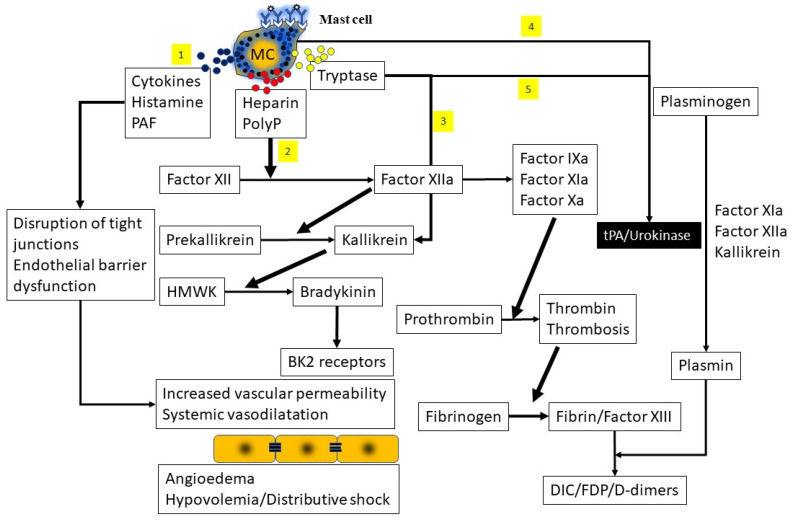
Activation of the contact system results in inflammatory responses, coagulation cascades with vascular thrombosis, fibrinolysis and complement activation. 1 = Mast cell derived histamine, PAF and IL-6 can cause disruption of tight and adherens junctions. 2 = mast cell derived heparin or PolyP can activate Factor XII which sequentially activates kallikrein and high molecular weight kininogen (HMWK), resulting in formation of bradykinin (BK) which can bind to BK receptors and increase vascular permeability. 3 = tryptase from mast cells could activate factor XII and kallikrein. 4 = Mast cells can activate fibrinolysis by secretion of tissue plasminogen activator (tPA) or urokinase. 5 = Mast cell tryptase has also been shown to activate fibrinolytic pathways. The net effect of contact pathway activation is increased capillary permeability, angioedema, and hypovolemic shock.

Activation of the contact system (Figure 8) results in inflammatory responses, coagulation cascades with vascular thrombosis, fibrinolysis and complement activation [26,95,96,97]. The contact system can be activated by artificial surfaces, misfolded proteins, RNA or DNA molecules, neutrophil extracellular traps (NETS), oversulfated chondroitin sulfate (OSCS) and mast cell-derived heparin (highly sulfated polysaccharide) and inorganic polymer (PolyP- also elaborated by platelets). Mast cell-derived heparin, released in anaphylactic reactions, provides a negative charged surface, leading to factor XII activation (factor XIIa). Heparin also binds antithrombin, which in turn irreversibly inhibits factor XIIa in a negative feedback loop [98,99]. Mast cell granules express the negatively charged chondroitin sulfate E, also capable of activating factor XII. Factor XII (Hageman factor) is a serine protease (serine endopeptidase that cleaves peptide bonds in proteins containing serine) that circulates as a zymogen (proenzyme) and undergoes a conformational change after contacting anionic surfaces and subsequently undergoing autoactivation. Factor XII has two substrates, Factor X1 and prekallikrein, both of which circulate complexed with human kallikrein (HK). Factor XIIa activates Factor XI, also a serine protease, which initiates the coagulation cascade, resulting in vascular thrombosis and the formation of fibrin clots [97]. Factor XIIa liberates kallikrein from plasma kallikrein which then cleaves FXIIa to β-FXIIa (a product that maintains proteolytic activity towards prekallikrein but not FXI) and high molecular weight kininogen (HMWK) to the nonapeptide bradykinin (BK). Bradykinin binds with high affinity to the BK2 receptor, resulting in the release of intracellular calcium, stimulation of endothelial nitric oxide synthase and mediating increased vascular permeability as well as edema and neutrophil chemotaxis. Binding of the receptor by BK results in the opening of endothelial tight junctions, fluid extravasation and hypovolemia. The receptor for BK is expressed in multiple cell types (endothelium, fibroblasts, epithelium, and smooth muscle) and can be induced by cytokines and hormones. A breakdown product, Des-Arg9-BK, resulting from the degradation of BK by carboxypeptidase 1, binds to BK1 receptor, which is upregulated in trauma and inflammation and is thought to be responsible for bronchoconstriction and hypotension seen in these reactions.

How is the contact system activation related to anaphylaxis? Deficiencies of FXII, HMWK, plasma kallikrein and BK2 receptor led to decreased IgE-mediated anaphylactic reactions in mice and a lower incidence of hypotension [95]. Contact system activation was shown in reactions to Chinese heparin contaminated with OSCS [100,101,102]. As stated earlier, mast cell-derived heparin has been shown to activate the contact system and may play a role in the hypotension and disseminated intravascular coagulation (DIC) seen in severe anaphylactic reactions [99,103]. Inal and colleagues described severe complications of anaphylaxis, including hypotension, thrombocytopenia, disseminated intravascular coagulation and pulmonary edema following administration of latex plasma expanders in a patient. The patient demonstrated strongly positive skin tests to latex with wheal and flare formation suggesting this might have been an IgE-mediated reaction [104]. Choi and colleagues similarly demonstrated thrombocytopenia, prolongation of prothrombin time, low fibrinogen levels but elevated degradation products, pulmonary congestion and renal hemorrhage in a mouse model of anaphylaxis [105]. These changes also followed injections of PAF and were completely prevented by pretreatment by a PAF antagonist, suggesting pivotal roles for PAF in this process. 

Alteration of coagulation parameters was also shown by Lombardini and coworkers, who evaluated a patient following a wasp sting [106]. The authors reported on an unclottable activated partial thromboplastin time, a significant anti-Xa activity and low fibrinogen levels probably due to mast cell-derived heparin (binding to anti-thrombin) and tryptase (cleaving the alpha and beta chains of fibrinogen thereby inhibiting clot formation). The authors noted that tryptase, by activating the single-chain urinary-type plasminogen activator, can be responsible for excessive fibrinogenolysis and fibrinogen depletion. Similar changes in coagulation pathways have been described in anaphylaxis complicating pregnancy, following bee sting acupuncture and in chronic urticaria [107,108,109,110,111]. 

### 3.5. Complement-Mediated Reactions

Activation of complement by immune complexes can lead to the generation of anaphylatoxins (such as C3a and C5a) which can then bind to and activate complement receptors on mast cells. This interaction can lead to mast cell degranulation and release of mediators culminating in reactions that may be difficult to distinguish from IgE-mediated allergy [14,15,16,26]. There is a growing list of drugs capable of causing such reactions that have been reviewed recently [112]. Lipid incipients, micellar drugs, liposomes, nanoparticles, polyethylene glycol and cellulose membranes can lead to complement activation and binding to complement receptors on mast cells and macrophages, resulting in the release of histamine and PAF, culminating in anaphylaxis [112]. Presumed mechanisms include binding of activated complement products (anaphylatoxins) to complement receptors on mast cells, basophils, and other cell types. Mast cell activation leads to elaboration of lipid mediators, histamine, proteases, PAF and cytokines, compounded by mediator release from other cell types that can have direct effects on blood vessels, smooth muscle cells and endothelium. Pulmonary vasoconstriction and pulmonary thromboembolism lead to respiratory distress, hypoxemia, cardiac ischemia, and cardiac arrhythmias [112]. Clinical manifestations include angioedema, cardiogenic shock, hypotension, respiratory distress, fever, chills, diaphoresis, and loss of consciousness. 

### 3.6. Direct Mast Cell Activation

Direct activation of mast cells can occur with drugs that interact with a specific, newly described Mas-related G-protein coupled receptor member X2 (MRGPRX2), independent of the IgE-FcεRI pathway. Such reactions can also exacerbate by other co-factors such as exercise or medications including beta-blocker drugs, angiotensin-converting enzyme inhibitors, nonsteroidal anti-inflammatory agents, and hormones [16,93]. In this pathway, binding of a select group of drugs such as Icatibant, quinolones, neuromuscular blocking drugs (tubocurarine, mivacarium, artacarium and rocuronium) and of drugs expressing the THQ (tetrahydroisoquinolinne) motif directly to MRGPRX2 results in protein kinase A and PI_3_ kinase pathway activation, calcium release and degranulation [113]. Other drugs are also capable of direct mast cell activation, including vancomycin (mediator release through ill-defined mechanisms), nonsteroidal anti-inflammatory drugs (inhibition of COX1 enzyme and enhanced release of cysteinyl leukotrienes and prostaglandin D_2_ and decreased production of anti-anaphylactic PGE_2_) and opiates (via binding to opiate receptors) [26]. Complement degradation products (C3a and C5a) as well as Toll-like receptor ligands (iron nanoparticles) can also activate mast cells [113]. Mast cell disorders, which can be primary (clonal disorders such as systemic mastocytosis and monoclonal mast cell activation syndrome), secondary (activation of mast cells by IgE-mediated allergic reactions) or idiopathic (such as the idiopathic mast cell activation syndrome/MCAS, idiopathic urticaria and idiopathic anaphylaxis/IA) may also be associated with enhanced releasability and increased frequency and severity of anaphylactic events [36,114,115,116]. 

### 3.7. Hereditary Alpha-Tryptasemia (HAT) and Other Genetic Disorders

Hereditary alpha-tryptasemia is an autosomal dominant syndrome described recently. Elevated basal serum tryptase >8 ng/mL is a diagnostic feature. In 2016, Lyons et al., demonstrated increased copy numbers of the gene encoding alpha-tryptase (TPSAB1) in the affected families, leading to higher tryptase levels and hence more severe symptoms [117]. The extra copies of tryptase gene leads to elevated pro-alpha tryptase production. Most affected individuals are very symptomatic and present with multisystem complaints (including irritable bowel-like symptoms, hives, pruritus, fatigue, musculoskeletal complaints, and anaphylactic reactions). A higher prevalence of the syndrome is seen in clonal mast cell disorders and in patients with idiopathic anaphylaxis. The disorder appears to be quite common, occurring in up to 5% of the population [118]. Unprovoked anaphylaxis was noted in over 50% of the patients tested in one study, with patients also concomitantly demonstrating a wide variety of systemic complaints-such as gastrointestinal, pulmonary, cutaneous, cardiovascular, and neuropsychiatric disease [119]. 

Deficiencies of PAF-acetyl hydrolase have also been associated with severe forms of anaphylaxis [53,120]. Ribo and coworkers also described a patient with severe anaphylactic reactions to paper wasp venom. This patient has a mutation in the KARS gene, which encodes lysyl-tRNA synthetase (LysRS), a protein that influences antigen dependent-FcεRI activation in mast cells [121]. The authors demonstrated constitutive activation of the microphthalmia transcription factor, involved in mast cell mediator synthesis and granule biogenesis. 

### 3.8. Idiopathic/Unknown Mechanisms

It is estimated that 30–60% of patients presenting with anaphylaxis may have no obvious etiological trigger to explain the disease and hence are described as having idiopathic anaphylaxis (IA). Recent advances in pathophysiology have delineated novel etiologies, including mast cell activation disorders, hereditary hyper-tryptasemia, hormone sensitivity syndromes (including catamenial anaphylaxis) and allergy to alpha-gal that need to be excluded before a diagnosis of IA is made. 

## 4. Conclusions

Anaphylaxis is a serious and potentially fatal allergic reaction arising from a multitude of causes. Major advances have been made in the understanding of endotypes (mechanisms) and of the role of mast cells, IgE, inflammatory mediators and cytokines in such reactions-especially at the endothelial level. There is an overwhelming need to develop specific, targeted therapies that prevent, or reverse capillary leak and distributive shock, which should be based on our molecular understanding of these processes. The role of endothelial apoptosis, the effects of histamine and PAF on endothelial gene expression, and the use of pharmacological agents targeting signaling molecules and transcription factors (other than adrenaline, antihistamines, and steroids) have not been studied in much detail in anaphylaxis. Lacking also are studies on the roles of gap and tight junctions, and specific molecules such as nectins, PECAM and CD99 in anaphylaxis. The development of biomarkers and radiological techniques to evaluate alterations in endothelial permeability in human anaphylaxis can greatly enhance management of severe allergic reactions and other capillary leak disorders.

## Figures and Tables

**Table 1:** Anaphylaxis by Endotype (Mechanism) and Phenotype (Presentation).

Endotype/Mediators	Phenotype/Symptoms	Triggers	Examples
**Immune-mediated**			
**IgE-dependent reactions** Mast cells, histamine, tryptase, platelet activating factor, nitric oxide, leukotrienes, proteases (chymase), prostaglandins, TNF-α	Urticaria, angioedema, vomiting, diarrhea, hypotension	Foods	Peanut, wheat, soy, milk, egg, shellfish
		Drugs	Antibiotics, anesthetics, chemotherapy drugs (platins, taxanes), biologics
		Alpha-Gal	Mammalian meat
		Hormones	Progesterone
		Hymenoptera venom	Wasps, Hornets
**IgG-mediated reactions** IgG immune complexes, serotonin, PAF synthesis, thromboxane A2	Wheezing, hypovolemia, hypotension, angioedema, diarrhea	IVIG	IgG/IgE anti-IgA antibodies (Common variable immune deficiency, IgA deficiency)
		Blood transfusion	IgA deficiency
		Immune compounds	Complement/immune complexes
		Monoclonal antibodies	Rituximab
**Cytokine release and cytokine storm reactions** (T-cell, TNF-α, IL-1β, IL-6, and MCP-1)	Flushing, nausea, chills, fever, hypoxemia, hypotension.	Chemotherapy drugs (cytokine release)	
		Monoclonal antibodies (cytokine storm)	
**Contact system activation** Factor XII-kallikrein-bradykinin activation, bradykinin receptors, endothelium	Angioedema, wheezing, hypotension	Drugs	Heparin and oversulfated chondroitin sulfate
**Complement-mediated reactions** Anaphylatoxins-Factors C5a/C3a, mast cell complement receptors, inflammatory pathway activation and cellular recruitment		Drugs	Protamine (heparin antidote)
		Membrane	Hemodialysis
		Toxins	Vespid Toxin (also IgE mediated)
		Vehicle	Polyethylene glycol
**Non-immune mediated (Idiopathic)**			
**Direct mast cell/basophil activation** Tryptase, cytokine, histamine release from mast cells/basophils	Angioedema, flushing, wheezing, hypotension	Aspirin and Nonsteroidal anti-inflammatory drugs	
		Opiates	
		Physical	Exercise, sunlight, temperature changes
		Media	Radiocontrast dyes
		Antibiotics and anesthetics	Fluoroquinolones, vancomycin, tubocurarine, atracuronium
**Primary Mast Cell Disorders**		Systemic mastocytosis	Exercise, drugs, hymenoptera venom
		Mast cell activation syndrome	Food, stress, alcohol
**Hereditary Alpha-Tryptasemia syndrome** Autosomal dominant syndrome	Diarrhea, myalgia, fatigue, urticaria, pruritus and anaphylaxis	Increased copy numbers of the gene encoding alpha-tryptase (TPSAB1)	Molecular testing confirms the diagnosis Droplet digital PCR testing of buccal samples
**Idiopathic anaphylaxis**	Urticaria, angioedema, hypotension, syncope, anaphylaxis	Exclude mast cell disorders, mammalian meat allergy and Hereditary alpha-tryptasemia	Diagnosis of exclusion
